# Downregulation of Sirtuin 1 Does Not Account for the Impaired Long-Term Potentiation in the Prefrontal Cortex of Female APPswe/PS1dE9 Mice Modelling Alzheimer’s Disease

**DOI:** 10.3390/ijms24086968

**Published:** 2023-04-09

**Authors:** Cátia R. Lopes, Joana S. Silva, Joana Santos, Matilde S. Rodrigues, Daniela Madeira, Andreia Oliveira, Ana Moreira-de-Sá, Vanessa S. Lourenço, Francisco Q. Gonçalves, Henrique B. Silva, Ana Patrícia Simões, Anabela P. Rolo, Paula M. Canas, Ângelo R. Tomé, Carlos M. Palmeira, João Pedro Lopes, Rodrigo A. Cunha, Paula Agostinho, Samira G. Ferreira

**Affiliations:** 1 CNC—Center for Neuroscience and Cell Biology, University of Coimbra, 3004-504 Coimbra, Portugal; 2 Department of Life Sciences, Faculty of Sciences and Technology, University of Coimbra, 3004-531 Coimbra, Portugal; 3 Faculty of Medicine, University of Coimbra, 3004-504 Coimbra, Portugal

**Keywords:** Alzheimer’s disease, APP/PS1 mice, memory, LTP, sirtuins, prefrontal cortex, synapse

## Abstract

Alzheimer’s disease (AD), which predominantly affects women, involves at its onset a metabolic deregulation associated with a synaptic failure. Here, we performed a behavioral, neurophysiological and neurochemical characterization of 9-month-old female APPswe/PS1dE9 (APP/PS1) mice as a model of early AD. These animals showed learning and memory deficits in the Morris water maze, increased thigmotaxis and anxiety-like behavior and showed signs of fear generalization. Long-term potentiation (LTP) was decreased in the prefrontal cortex (PFC), but not in the CA1 hippocampus or amygdala. This was associated with a decreased density of sirtuin-1 in cerebrocortical synaptosomes and a decreased density of sirtuin-1 and sestrin-2 in total cerebrocortical extracts, without alterations of sirtuin-3 levels or of synaptic markers (syntaxin, synaptophysin, SNAP25, PSD95). However, activation of sirtuin-1 did not affect or recover PFC-LTP deficit in APP/PS1 female mice; instead, inhibition of sirtuin-1 increased PFC-LTP magnitude. It is concluded that mood and memory dysfunction in 9-month-old female APP/PS1 mice is associated with a parallel decrease in synaptic plasticity and in synaptic sirtuin-1 levels in the prefrontal cortex, although sirtiun1 activation failed to restore abnormal cortical plasticity.

## 1. Introduction

Alzheimer’s disease (AD) is a progressive neurodegenerative disease, which is significantly more common in women than in men [1]. AD is clinically characterized by memory loss and cognitive deficits together with cerebral cortical thinning and loss of hippocampal volume [2,3]. Neuropathology characterizes AD by the extracellular accumulation of β-amyloid (Aβ) peptides in plaques and hyperphosphorylation of tau in neurofibrillary tangles intracellularly [2,3]. Synaptic dysfunction, namely of excitatory synapses [4,5] appears early at the onset of the disease, and it is the best correlator with cognitive deficits [6]. Additionally, metabolic deregulation, typified by impaired glucose utilization, is also associated with the onset of cognitive deficits, as heralded by the utilization of deoxyglucose PET imaging as ancillary evidence to diagnose AD [7] and the increased awareness that metabolic deregulation is tightly linked to synaptic dysfunction [6].

Amongst the major intracellular metabolic coordinators, increasing interest is devoted to sirtuins, a family of NAD^+^-dependent histone deacetylases (HDACs) with a parallel role in the control of epigenetic as well as stress-adaptive metabolic processes [8]. This stems from the pioneering work demonstrating that dampening sirtuin 1 (SIRT1) is detrimental to synaptic plasticity and memory [9,10]; this might involve a parallel transcriptional control together with a metabolic re-adaptation, in line with the localization of SIRT1 mainly in the nucleus, but also in the cytosol, in particular in synapses [11,12], regulating mitochondrial dynamics and function, contributing to format antioxidant and stress responses and regulating metabolic pathways, such as gluconeogenesis and fatty acid metabolism [13,14]. Several studies have documented a relation between sirtuins and AD, as typified by the proposed association of polymorphisms of SIRT1 with AD [15,16] and the decrease in the levels of SIRT1 and SIRT3 in AD patients [17,18] and animal models [19,20]. Accordingly, different strategies to bolster SIRT1 ameliorate the deficits of synaptic plasticity and of learning and memory in animal models of AD [21,22,23]. However, some conflicting evidence questions the relation between the levels and activity of sirtuins and functional deterioration in AD [24,25].

We now used 9-month-old female APPswe/PSEN1dE9 (abbreviated APP/PS1) mice, which harbor the APP human Swedish mutation and a presenilin delta 9 mutation [26], to model early AD pathology. These transgenic APP/PS1 mice show synaptic and memory deficits starting at 5 months and Aβ_42_ plaques as early as 6 months of age, all evolving up to 9–12 months of age (e.g., [27,28,29]). After defining the presence of different memory-related behavioral deficits, we characterized the alterations of synaptic plasticity in different brain regions to explore their association with alterations of the level and activity of sirtuins. We report that mood and memory dysfunction in 9-month-old female APP/PS1 mice is associated with a parallel decrease in synaptic plasticity and in synaptic SIRT1 levels in the prefrontal cortex, although SIRT1 activation failed to restore this abnormal cortical plasticity.

## 2. Results

### 2.1. Female APP/PS1 Mice 9-Month-Old Display Mood and Memory Deficits

**Figure 1:**
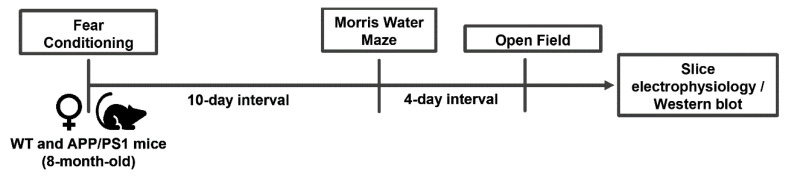
Overall schematic presentation of the experimental protocol.

**Figure 2:**
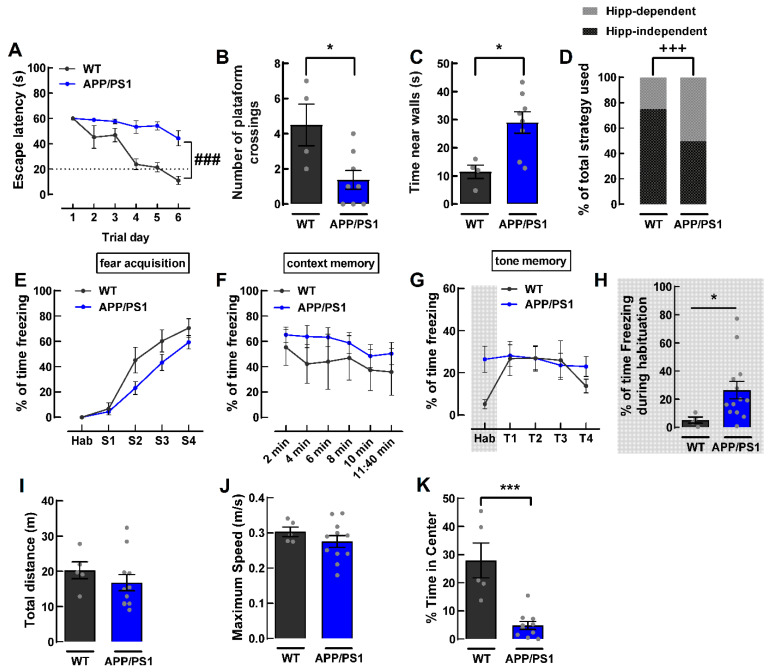
Behavioral characterization of 8–9-month-old female APP/PS1 mice. (**A**) Acquisition/learning in the Morris water maze (MWM) test was assessed by measuring the latency to reach the platform during the 6 days of the test; APP/PS1 mice displayed learning deficits. (**B**) Number of crossings in the platform site in the probe day of the MWM, showing reference memory deficits in APP/PS1 mice. (**C**) Time spent swimming close to the walls in the MWM, showing that APP/PS1 mice did more thigmotaxic swim. (**D**) Percentage of animals that used a hippocampus-dependent versus a non-hippocampus dependent search strategy for the platform during the probe trial in the MWM; APP/PS1 mice used significantly less hippocampus-dependent strategies than the WT mice. There was no difference in fear acquisition and memory, as shown in: (**E**) fear acquisition in the cued (tone) fear conditioning test represented as the percentage of time freezing; (**F**) Percentage of time freezing in the same context 24 h post-acquisition; and (**G**) percentage of time freezing upon presentation of the same tone, but in a different context. (**H**) Percentage of time spent in freezing during habituation phase, showing increased freezing, suggestive of fear generalization. (**I**) There was no difference in locomotion, as total distance travelled in the open field and (**J**) the maximum speed reached in meters by seconds were similar between APP/PS1 and WT mice. (**K**) Percentage of time spent in the center of the open field, showing increased anxiety-like behavior in APP-PS1 mice. Data are mean ± SEM of 4–13 animals. ### *p* < 0.001, by a Two-way ANOVA test; * *p* < 0.05 and *** *p* < 0.001 by a Student’s unpaired *t*-test; +++ *p* < 0.001, Chi-square test.

Female APP/PS1 mice were specifically selected for this study since AD is more frequent in women [1] and female mice models of AD have been less explored than males. Female APP/PS1 mice were first characterized by behavior analysis for a confirmation of their AD-like phenotype (Figure 1), which could only be consistently observed from 9 months of age onwards. We assessed spatial learning and spatial reference memory by using the Morris water maze (MWM) test. In the spatial acquisition phase, we found that the APP/PS1 mice took significantly more time to reach the platform when compared to their wild type littermates (F_5,60_ = 5.102, *p* = 0.0006, *n* = 4–8, Figure 2A), which indicates a deficit in spatial learning, as expected. In fact, APP/PS1 mice never reached the criterion (latency of 20 s to reach the platform) for learning. On the probe day, 24 h after the last spatial acquisition day, APP/PS1 mice displayed deficits in spatial reference memory as they crossed the site of the platform fewer times than WT mice (WT: 4.5 ± 1.2; APP/PS1: 1.4 ± 0.5; *p* = 0.0183, *n* = 4–8, Figure 2B). MWM performance can be affected by thigmotaxic behavior (swimming close to the walls), which may indicate increased anxiety-like behavior [30] or impairment in the choice of optimal strategies to locate the platform [31]. Therefore, we also evaluated the time spent in thigmotaxic swim. APP/PS1 mice spent significantly more time swimming close to walls than control mice (WT: 11.4 ± 2.4 s; APP/PS1: 29.0 ± 3.8 s; *p* = 0.0121, *n* = 4–8, Figure 2C), indicating an increased anxiety-like behavior or deficits in the choice of optimal strategies in APP/PS1 mice as compared to WT mice. When evaluating the pattern of search strategies used to find the location of the platform on the probe day [31], we observed that most WT mice used hippocampus-dependent strategies (75%), a value significantly higher than that observed for APP/PS1 mice, which used non-hippocampus-dependent and hippocampus-dependent strategies equally (50%) (*p* = 0.0003, *n* = 4–8, Figure 2D).

To assess associative learning, we used the fear conditioning test, which relies on an association between a neutral conditioned stimulus (tone) and a noxious unconditional stimulus (foot shock) [32]. Mice are expected to exhibit a conditioned freezing response, a form of defensive behavior [32]. We did not observe significant differences between WT and APP/PS1 mice in fear acquisition in response to foot shock (F_4,64_ = 1.800, *p* = 0.1397, *n* = 5–13, Figure 2E). After 24 h, we evaluated memory to the context where the same conditions for the acquisition were kept, except that no tone or shock was delivered. There was no difference in contextual fear memory between WT and APP/PS1 mice, as they spent a similar percentage of time freezing when exposed to the same context used during acquisition (F_5,80_ = 0.3361, *p* = 0.8897, *n* = 5–13, Figure 2F). On the third day of the test, we evaluated the memory to the tone in a different context. APP/PS1 mice displayed a higher percentage of time spent freezing than control mice in the habituation phase of the test (WT: 5.17 ± 2.18%; APP/PS1: 26.42 ± 6.20%; *p* = 0.0101, *n* = 4–13; Figure 2G,H). This is likely to be an indicator that APP/PS1 generalizes fear to other contexts. However, upon tone presentation, there was no difference between APP/PS1 mice and their WT littermates (F_3,45_ = 0.7862, *p* = 0.5079, *n* = 4–13, Figure 2G). This suggests that 9-month-old female APP/PS1 mice do not have impairments in tone fear memory.

We further controlled for differences in locomotion and anxiety-like behavior by using the open field test. We found no differences in locomotion between the WT and APP/PS1 mice, as there was no significant difference in the total distance travelled in the apparatus (WT: 20.29 ± 2.40 m, APP/PS1: 16.77 ± 2.30 m, *p* = 0.3714, *n* = 5–11, Figure 2I). There was also no difference in the maximum speed reached (WT: 0.30 ± 0.01 m/s, APP/PS1: 0.28 ± 0.02 m/s, *p* = 0.328, *n* = 5–11; Figure 2J). However, we found that APP/PS1 mice displayed higher anxiety-like behavior when compared to the WT mice, as the latter spent more time in the center of the open field (WT: 27.91 ± 6.20%, APP/PS1: 4.85 ± 1.44%, *p* = 0.0003, *n* = 5–11, Figure 2K).

A summary of the behavior alterations observed in 9-month-old female APP/PS1 mice is presented in Table 1.

### 2.2. Lower Magnitude of LTP in the PFC of 9-Month-Old Female APP/PS1 Mice

**Figure 3:**
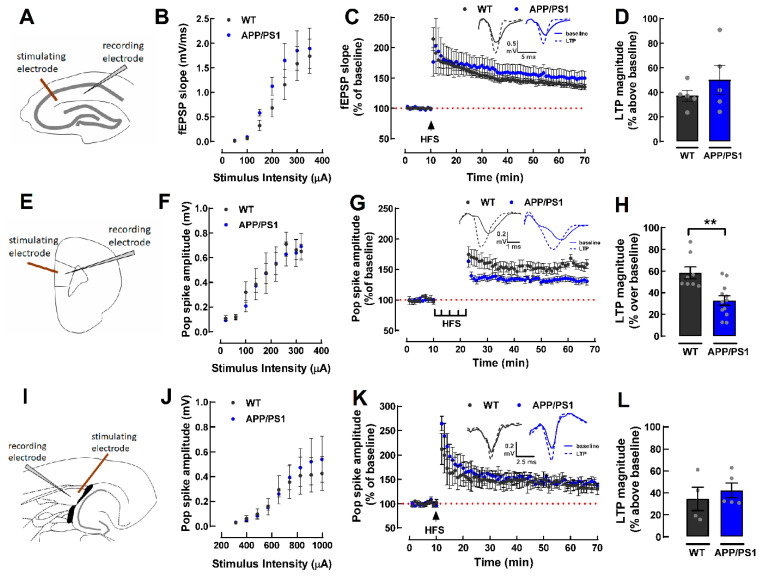
Synaptic transmission and plasticity in the dorsal hippocampus, prefrontal cortex, and amygdala of 9-month-old female WT and APP/PS1 mice. In the dorsal hippocampus, synaptic transmission was measured in Schaffer fibers-CA1 pyramid synapses (**A**) by measuring the slope of field excitatory postsynaptic potentials (fEPSP). WT and APP/PS1 mice displayed similar input/output response (**B**) and similar magnitude of long-term potentiation (LTP), triggered by a high-frequency train (HFS, 100 Hz for 1 s) (**C**,**D**). In the prelimbic prefrontal cortex (PFC), synaptic transmission between pyramidal neurons in layer II/III and layer V (**E**) was measured as the amplitude of the population spike (PS) responses. WT and APP/PS1 mice displayed similar input/output response (**F**), but relative to the WT, APP/PS1 mice displayed a decreased LTP magnitude, triggered by a high-frequency train (HFS, 5 trains of 300 pulses at 100 Hz every 3 min) (**G**,**H**). Inside the lateral amygdala (**I**), synaptic transmission was measured as the amplitude of the PS responses. WT and APP/PS1 mice displayed similar input/output response (**J**) and similar LTP magnitude, triggered by a high-frequency train (HFS, 3 trains of pulses of 100 Hz delivered every 5 s) (**K**,**L**). Data are mean ± SEM of 4–12 experiments (number of different animals tested). ** *p* < 0.01, Student’s unpaired *t*-test.

Based on the results from the behavior tests, we evaluated synaptic plasticity in key structures involved in learning and memory, as well as emotional processing, namely the dorsal hippocampus, the PFC and the amygdala. In slices from the dorsal hippocampus (Figure 3A), we did not observe differences neither in basal synaptic strength (Figure 3B), nor in the magnitude of LTP between WT and APP/PS1 (WT: 37.24 ± 4.50% and APP/PS1: 50.19 ± 11.95% change over baseline, *p* = 0.3392, *n* = 5, Figure 3C,D). In slices from the PFC (Figure 3E), although there was no difference in basal synaptic strength (Figure 3F), the magnitude of LTP was lower in APP/PS1 as compared to the WT mice (WT: 58.33 ± 5.44% and APP/PS1: 32.76 ± 4.31% change over baseline, *p* = 0.0016, *n* = 8–12, Figure 3G,H). Regarding the amygdala (Figure 3I), there was also no difference either in the basal synaptic strength (Figure 3J) or in the magnitude of LTP (WT: 34.58 ± 10.66% and APP/PS1: 42.25 ± 6.68% change over baseline, *p* = 0.5444, *n* = 4–5 Figure 3K,L).

### 2.3. Lack of Alterations of Synaptic Markers in Cortical Synaptosomes of APP/PS1 Mice

**Figure 4:**
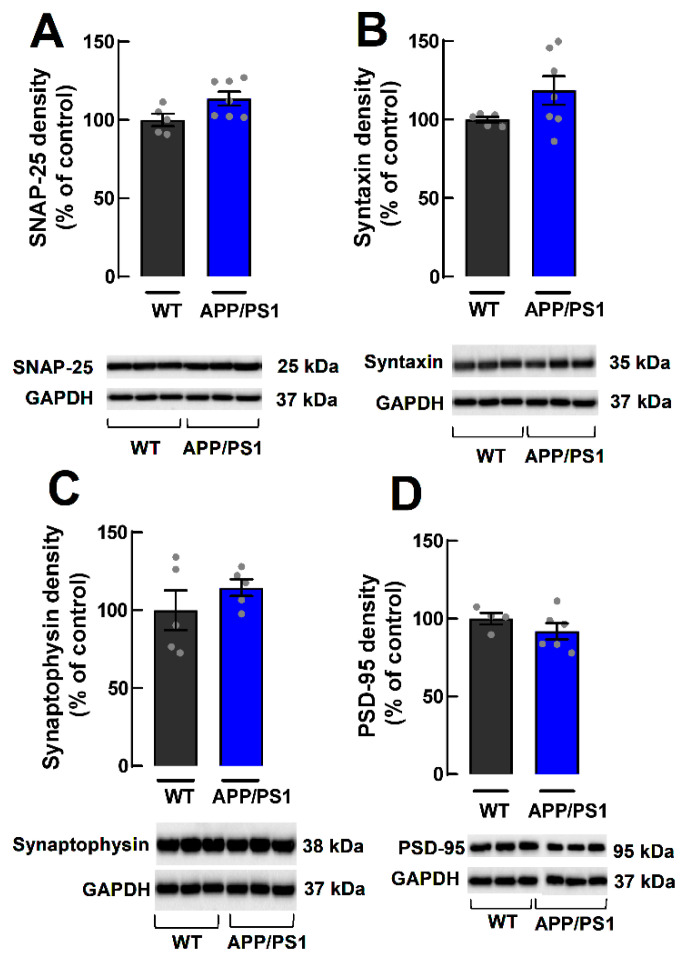
Relative density of synaptic markers in cerebrocortical synaptosomes of 9-month-old female WT and APP/PS1 mice. There were no significant differences in the density of the synaptic proteins evaluated, as shown in (**A**) SNAP-25, (**B**) syntaxin, (**C**) synaptophysin and (**D**) PSD-95. Data are mean ± SEM of 4–7 experiments (number of different animals tested).

Loss of synaptic markers in the hippocampus is an early feature of AD and is correlated with cognitive decline [6]. Given the lower LTP in the PFC, we used Western blotting to assess, in cortical synaptosomes (purified synapses), the density of different synaptic markers, namely the presynaptic markers SNAP-25 and syntaxin, the general marker of synaptic vesicles synaptophysin and the postsynaptic density marker of excitatory synapses, PSD-95 (Figure 3A–D). We found no alterations in the density of SNAP-25 (WT: 100.00 ± 3.88%, APP/PS1: 113.60 ± 4.39%; *p* = 0.0520, *n* = 5–7, Figure 4A), syntaxin (WT: 100.00 ± 1.75%, APP/PS1: 118.40 ± 9.15%, *p* = 0.1276, *n* = 5–7, Figure 4B), synaptophysin (WT: 100.00 ± 12.75%, APP/PS1: 114.50 ± 5.47%, *p* = 0.3270; *n* = 5; Figure 4C) or PSD-95 (WT: 100.00 ± 3.74%, APP/PS1: 91.94 ± 5.09%, *p* = 0.2835, *n* = 4–6, Figure 4D). These results indicate that at 9 months of age, female APP/PS1 mice do not display a loss of synaptic proteins in cerebrocortical synapses.

### 2.4. Decreased SIRT1 in Cerebrocortical Synapses from 9-Month-Old Female APP/PS1 Mice

**Figure 5:**
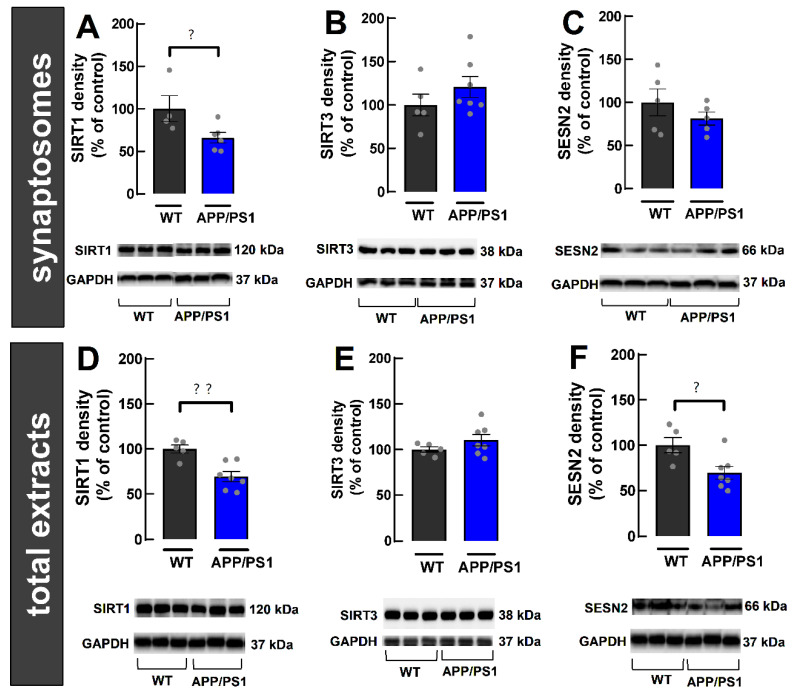
Alteration of the relative density of intracellular metabolic controllers in synapses (**A**–**C**) and outside synapses (**D**–**F**) in the cerebral cortex of 9-month-old female WT and APP/PS1 mice. (**A**–**C**) Cerebrocortical synaptosomes (purified synapses) from APP/PS1 mice displayed a decreased density of sirtuin 1 (SIRT1; (**A**)), whereas there was no change in the density of sirtuin 3 (SIRT3; (**B**)) or of sestrin 2 (SESN2; (**C**)). (**D**–**F**) In total extracts (mostly extra-synaptic content) from APP/PS1 mice, there was a decreased density of SIRT1 (**D**) and of SESN2 (**F**), whereas there was no change in the density of SIRT3 (**E**). GAPDH (1:5000) was used as a loading control and results are presented as a ratio between the density of the protein of interest and GAPDH. Data are mean ± SEM of 4–7 experiments (number of different animals tested). ? *p* < 0.05 and ?? *p* < 0.01, using a Student’s unpaired *t*-test.

Since metabolic deregulation is tightly linked to synaptic dysfunction [6], we further used synaptosomes from the cerebral cortex to evaluate the densities of some key intracellular controllers of cellular metabolism, namely sirtuins 1 and 3 (SIRT1 and SIRT3) and sestrin 2 (SESN2). SIRT1 density was decreased in cerebrocortical synaptosomes (WT: 100.00 ± 15.46%, APP/PS1: 66.09 ± 6.12%, *p* = 0.0466, *n* = 4–6, Figure 5A) of APP/PS1 compared to WT mice. In contrast, we did not find alterations in SIRT3 density (WT: 100.00 ± 12.46%, APP/PS1: 120.70 ± 11.99%, *p* = 0.2699, *n* = 5–7, Figure 5B). As for SESN2 density, we found no changes (WT: 100.00 ± 15.54%, APP/PS1: 81.29 ± 7.70%, *p* = 0.3123, *n* = 5, Figure 5C). Overall, our results show a decrease in the density of SIRT1 specifically in cerebrocortical synapses from 9-month-old APP/PS1 female mice.

### 2.5. Decreased Density of SIRT1 and SESN2 in Total Extracts from the Cerebral Cortex of 9-Month-Old Female APP/PS1 Mice

We then used total extracts from the cerebral cortex to evaluate the same metabolic controllers as above. The goal is to assess if their densities are altered in the cytosol of other cellular components, including in neurons, but outside of synapses, as well as in astrocytes and microglia, which form the bulk of total extracts, given that synapses only represent circa 1–2% of cortical tissue [33]. We found that SIRT1 density is decreased (WT: 100.00 ± 4.64%, APP/PS1: 69.3± 5.57%, *p* = 0.0026; *n* = 5–7, Figure 5D) in APP/PS1 mice compared to WT mice. In contrast, SIRT3 density was not altered (WT: 100.00 ± 2.87%; APP/PS1: 110.30 ± 6.37%; *p* = 0.2284; *n* = 5–7; Figure 5E). As for SESN2 density, we observed a decrease in APP/PS1 compared to WT mice (WT: 100.00 ± 8.44%, APP/PS1: 69.76 ± 7.03%, *p* = 0.0202, *n* = 5–7, Figure 5F). Overall, our results suggest global changes in the cerebral cortex of female 9-month-old APP/PS1 mice, involving a decreased density of SIRT1 and SESN2. These alterations have the potential to impair cellular metabolism.

### 2.6. Pharmacological Activation of SIRT1 Does Not Recover LTP Deficit in PFC Slices from APP/PS1 Mice

**Figure 6:**
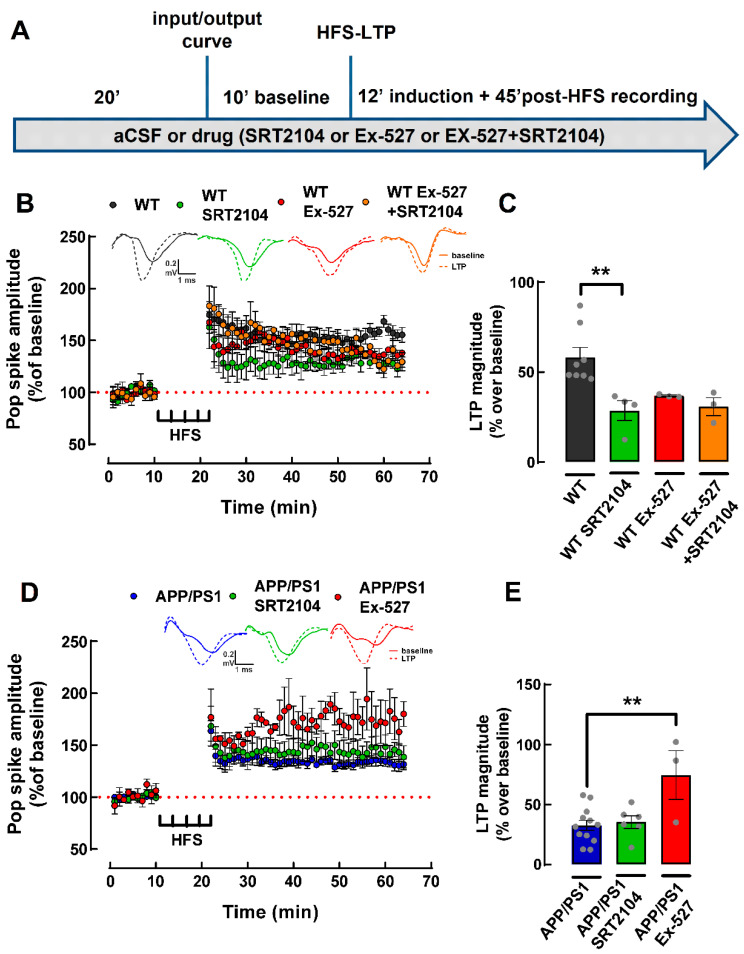
Effect of the pharmacological activation and inhibition of SIRT1 on PFC-LTP in 9-month-old WT and APP/PS1 mice. (**A**) Scheme of the experimental protocols illustrating the timing of addition of the different tested drugs in the electrophysiological experiments. (**B**) Time course and (**C**) summary graph showing that activation of SIRT1 with SRT2104 (10 µM) decreased the magnitude of LTP, while inhibition of SIRT1 with Ex-527 (1 µM) showed a tendency (*p* = 0.0896) to decrease the magnitude of LTP in the PFC of WT mice. There was no effect of SRT2104 on top of Ex-527. (**D**) Time course and (**E**) summary graph showing that activation of SIRT1 with SRT2104 did not affect LTP magnitude, while SIRT1 inhibition with Ex-527 significantly increased the magnitude of LTP in the PFC of APP/PS1 mice. Data are mean ± SEM of 3–12 experiments (number of different animals tested). ** *p* < 0.01, one-way ANOVA test.

SIRT1 is important for synaptic plasticity, cognition and learning [9,10]. Therefore, we used the SIRT1 activator SRT2104 (10 µM; [34]) to evaluate whether activating SIRT1 would recover the LTP deficit observed in the PFC of APP/PS1 female mice. A one-way ANOVA analysis revealed that SRT2104 decreased the magnitude of PFC-LTP in WT mice (WT: 58.33 ± 5.44%, WT+SRT2104: 28.67 ± 5.46% change over baseline, *p* = 0.0064, *n* = 4–8, Figure 6A,B). However, there was also a tendency for a decreased PFC-LTP magnitude in WT mice in the presence of the SIRT1 inhibitor Ex-527 (1 µM; [35]) (WT+Ex-527: 36.89 ± 0.49% change over baseline, *p* = 0.0896 when compared to WT, *n* = 3–8, Figure 6A,B). In the presence of the SIRT1 inhibitor, no additional effect of the SIRT1 activator was observed in WT mice (WT+Ex-527+SRT2104: 30.90 ± 4.91% change over baseline, *p* = 0.9639 when compared to the WT+Ex-527, *n* = 3; Figure 6A,B). Surprisingly, no effect of SRT2104 was not observed in PFC-LTP magnitude in APP/PS1 mice (APP/PS1: 32.76 ± 4.31%, APP/PS1+SRT2104: 35.45 ± 5.16% change over baseline, *p* = 0.9432, *n* = 6–12 Figure 6C,D), meaning that exogenous SIRT1 activation does not recover LTP deficits in the PFC of 9-month-old APP/PS1 female mice. Instead, there was a significant increase in the magnitude of LTP when the SIRT1 inhibitor Ex-527 was present (APP/PS1+Ex-527: 74.71 ± 20.27% change over baseline, *p* = 0.0036 when compared to APP/PS1, *n* = 3–12; Figure 6C,D), implying that the role of SIRT1 may be altered in association with memory impairment, although bolstering SIRT1 activity does not recover PFC-LTP deficits in 9-month-old APP/PS1 female mice.

## 3. Discussion

AD is a neurodegenerative disease that predominantly affects women [1], which is still without any available therapeutic prospect in view of our incomplete knowledge of its etiology. Based on the accumulating evidence indicating that synaptic dysfunction and metabolic deregulation are two core features of the onset of AD (reviewed in [6]), we now explored in females of a purported AD mouse model the relation between memory deficits, alterations of synaptic plasticity and of intracellular metabolic coordinators. We found that 9-month-old female APP/PS1 mice displayed deficits in learning, memory and mood-related behavior, associated with a decreased LTP magnitude in the prefrontal cortex (PFC). Notably, this deficit of PFC-LTP was not associated with the loss of synaptic markers, but instead with decreased levels of the metabolic regulator, sirtuin 1 (SIRT1). However, the activation of SIRT1 failed to correct the abnormal PFC-LTP in APP/PS1 female mice, which casts doubts on the therapeutic potential of targeting SIRT1 to ameliorate memory dysfunction in female AD mice.

The behavioral characterization of female APP/PS1 mice only revealed clear deficits of learning and memory in the MWM at 9 months of age (see also [36]), somewhat later than male APP/PS1 mice, which display memory deficits at 5–7 months of age in our hands [29] and in the hands of others (e.g., [37,38,39]). These deficits of spatial reference memory, which were re-enforced by the alteration in the search strategy utilized by APP/PS1 female mice in the Morris water maze, were not accompanied by deficits of fear memory, which have been reported in some studies using male APP/PS1 mice (e.g., [28,36,40,41]) but not in others [42,43,44]. Instead, APP/PS1 female mice displayed an apparently increased fear generalization and an anxiogenic profile concluded by a thigmotaxic behavior in the Morris water maze and a decreased time spent in the center of an open field. The suggestion of increased fear generalization in APP/PS1 female mice was based on the observed increased freezing during habituation in a different context (day 3; see [45]) and is in notable agreement with the higher incidence of fear-related disorders in AD patients [46] and with the suggestion that fear-based neuropsychiatric disorders may be an early marker of AD (see [47,48]). Overall, our behavioral analysis of 9-month-old female APP/PS1 mice indicates the presence of spatial reference memory deficits accompanied by mild emotional alterations. 

Based on the prominent role of the hippocampus, in particular of hippocampal synaptic plasticity, in processing spatial reference memory (e.g., [49]), it was surprising to observe a lack of modification of the pattern of hippocampal LTP in female APP/PS1 mice. A similar lack of alteration of hippocampal LTP magnitude was also observed by others in male APP/PS1 mice [29,50] (but see [37,39,51]) but the possible clarification of a putative sex-relate difference will require a time-dependent direct comparison between sexes in the same sub-strain of APP-PS1 mice. Instead, we observed that female APP/PS1 mice displayed a decreased LTP magnitude in the PFC, a brain structure that is known to encode several behavioral features relevant to the performance of spatial memory tasks, such as goal location, decision making, strategy switch and weighted reward (e.g., [52,53]). Accordingly, PFC hypofunction is observed in AD patients (reviewed in [54]), where PFC-LTP-like deficits are predictive of AD [55], and animal models of AD also display functional and morphological alterations in excitatory synapses of the PFC (e.g., [56,57,58,59]). Based on the relation between the loss of synaptic markers and deficits of hippocampal synaptic plasticity accompanying memory deterioration in different animal models of aging and brain disease (e.g., [60,61,62]), it was surprising to conclude that there was no alteration in the levels of different synaptic markers (SNAP25, syntaxin, synaptophysin, PSD95) in cerebrocortical synapses of female APP/PS1 mice displaying memory deficits. In AD patients, there is a loss of synaptic markers in the PFC that correlates with memory deterioration [63,64,65]. However, female APP/PS1 mice at 9 month of age model the onset of AD and it is not known if a loss of synaptic markers in the PFC occurs at the onset of memory deterioration in AD, as is the case in the hippocampus in patients [66] (meta-analysis in [67]) or in animal models (e.g., [5,68,69,70]).

Since metabolic deregulation in synapses might also account for the deterioration of synaptic plasticity and memory performance (reviewed e.g., [71]) and there is compelling evidence that metabolic set up of prefrontocortical synapses is modified in animal models of AD [72], we explored the possibility that alterations of intracellular metabolic coordinators might account for the deterioration of PFC-LTP observed in female APP/PS1 mice. We focused on sirtuins since their levels are reduced in the hippocampus of AD patients [17,18] and animal models [19,20] and their genetic and pharmacological manipulation has been reported to ameliorate several features of AD in animal models [21,22,23]. We found that female APP/PS1 mice displayed a significant reduction in the levels of SIRT1 both in cortical synapses as well as in extrasynaptic cortical compartments. Indeed, SIRT1 levels decrease with AD progression in cortical homogenates of AD patients [17]. In the parietal cortex of AD patients, a decrease in SIRT1 and *Sirt1* mRNA levels was also found and correlated with Aβ and tau accumulation [24]. Moreover, SIRT1 was proposed as a predictive marker in AD patients, as its serum concentration decreases with age, and in a more pronounced way in MCI and AD patients [73]. In contrast to SIRT1, we found no alterations in the levels of SIRT3 in synapses and in the cerebrocortical homogenates of 9 months old female APP/PS1 mice. Previous studies reported a decrease in the levels of *sirt3* mRNA and protein density in the cerebral cortex of 12-month-old APP/PS1 mice [19] but not in the 6-month-old PDAPP mice [74]. In humans, *sirt3* mRNA levels and protein density in the cerebral cortex of AD patients were decreased compared to controls [75]. Thus, it is possible that at 9 months of age, the alterations in SIRT3 levels may not yet be present in our female APP/PS1 mice. Additionally, given the lack of studies using female animals, a sex-dependent change cannot be ruled out at this point. With respect to SESN2, which was proposed to have a neuroprotective role in AD [76], we found a decreased density in cerebrocortical extracts, supporting a decreased adaptability of cortical cells to various kinds of cellular stress in our model. However, our data contrast with previous data showing increased density in cortical lysates, although in 12-month-old APP/PS1 mice [77], and increased SESN2 mRNA levels in MCI and AD patients [78].

Given that the more consistent alteration was the decrease of SIRT1 density, in particular in cerebrocortical synapses, we tested if the pharmacological activation of SIRT1 could revert the LTP deficits observed in the PFC of APP/PS1 mice. We found that activating SIRT1 with SRT2104 decreased LTP-PFC in WT mice, which is in contrast with the findings that the genetic deletion of SIRT1 in the PFC resulted in decreased LTP [9,10]. However, inhibition of SIRT1 with Ex527 also resulted in decreased LTP magnitude, consistent with the literature showing that the endogenous activity of SIRT1 bolsters LTP [9]. This paradoxical similar effect of activators and inhibitors of SIRT1 probably results from the effect of different populations of SIRT1 located synaptically and extrasynaptically, in nuclear compartments, astrocytes or microglia, although such a contention will require future experimental confirmation. However, irrespective of a purported contribution of different populations of SIRT1, we observed that SIRT1 activation was not able to recover the LTP deficit present in the PFC in our 9-month-old APP/PS1 female mice. Instead, to our surprise, it was SIRT1 inhibition that increased PFC-LTP in APP/PS1 female mice. These are results to be further clarified in the future, including an evaluation of potential sex-dependent differences in the action of sirtuins that was identified in some recent studies [79,80,81].

In conclusion, we found that 9-month-old female APP/PS1 display impairments in learning, memory and mood-related behavior, that were associated with LTP deficits in the PFC, which could not be recovered upon SIRT1 activation. These findings cast doubts on the therapeutic usefulness of SIRT1 activators as candidates to control alterations in early AD, while prompting the quest for other systems controlling PFC-LTP that might be of interest to develop strategies to manage the onset of AD. As a final note, although we did not carry out a direct investigation of the impact of sex on the development of AD features, the comparison with previous reported findings in male APP/PS1 mice with the presently reported characteristics of the onset of behavioral, electrophysiological and neurochemical alteration in female APP/PS1 mice suggest the possibility of sex-related differences that are of particular relevance when considering the different incidence of AD in men and women.

## 4. Materials and Methods

### 4.1. Animals

Female APPswe/PSEN1dE9 (henceforward APP/PS1) transgenic mice with 5–9 months were initially purchased from The Jackson Laboratory (MMRRC stock #34832; donated by Dr. David R Borchelt, University of Florida). To maintain the colonies, male hemizygous APP/PS1 mice were crossed with female WT mice. In this study, 8–9 months old mice were obtained by crossing female WT (B6C3F1/J) mice with male hemizygous APP/PS1 mice. All mice were kept in cages with 1–3 littermates and with ad libitum food and water. The cages were kept at 21–23 °C with 50–60% humidity under a 12 h dark/light cycle with light from 7AM to 7PM. All procedures were carried out in accordance with the guidelines of the European Community guidelines (EU Directive 2010/63/EU) and the Portuguese law on animal care (1005/92) and approved by the Ethical Committee of the Center for Neuroscience and Cell Biology of (ORBEA n° 243_2020/1210-72020).

### 4.2. Drugs

The SIRT1 activator SRT2104 (4-methyl-N-[2-[3-(morpholin-4-ylmethyl)imidazo[2,1-b][1,3]thiazol-6-yl]phenyl]-2-pyridin-3-yl-1,3-thiazole-5-carboxamide) was purchased from MedChemExpress (Cat# HY-15262) and the SIRT1 inhibitor Ex-527 (6-chloro-2,3,4,9-tetrahydro-1H-carbazole-1-carboxamide, Sigma, E7034) was obtained from Tocris (Cat# 2780).

### 4.3. Behavioral Analysis

Behavioral analysis was performed as previously described [61] in a sound-attenuated room maintained at 21–23 °C and 50–60% humidity with red lightening (10 lux light intensity). Mice were habituated in the behavioral room for at least 1 h before beginning behavioral tests. Between trials, the apparatus and objects used were cleaned with a 10% ethanol solution. Animals from the same cage were kept in transport cages between trials to avoid contact between animals that had already completed the test and animals that had not. The tests were video-recorded and analyzed with the ANY-maze Video Tracking Software (Stoelting Europe, Dublin, Ireland). The test schedule was the following: (1) fear conditioning, (2) Morris water maze (MWM) starting 10 days after the end of the fear conditioning test, and (3) open field test, 4 days after the end of the MWM (see Figure 1).

Fear conditioning testing was carried out as previously described [82], with minor modifications. Briefly, in the acquisition phase, animals were placed in a sound-attenuated chamber and habituated for 140 s. The chamber had transparent PVC walls, was covered at the top and the floor was composed of grid metal (context A). The chamber was illuminated by white light (450–650 nm) and near-infrared light (940 nm). After the habituation, an auditory tone of 4 kHz, 80-dB was delivered for 20 s. In the last 2 s of the auditory tone, a 0.5-mA foot-shock was delivered. The auditory tone and foot-shock were repeated 4 times with a 120-s interval, totalling 4 auditory tones and 4 foot-shocks. The time mice spent in freezing behavior was evaluated, in the habituation before the shock and then between shocks, thus, for 128 s. Freezing is a species-specific defence mechanism that has been defined as the suppression of all movement except that required for respiration. To evaluate contextual fear conditioning, 24 h after the acquisition phase, the animals were placed again in the same chambers in context A, except that no auditory tone or foot-shock was delivered. The time spent in freezing behavior was evaluated in every minute of the test. To evaluate memory to the tone, 72 h after the acquisition phase, animals were placed in the sound-attenuated chamber in context B to avoid spatial recognition. Context B differed from context A in three dimensions: first, the walls of the chamber had a different pattern; second, the floor was covered with white PVC; third, the chamber was illuminated only by near-infrared light (940 nm). After the 140 s of habituation, a 4 kHz, 80-db auditory tone was delivered for 20 s. No foot-shock was delivered. Another auditory tone was delivered 120 s after, and the process was repeated a total of 4 times. Time spent in freezing behavior was evaluated immediately after each auditory tone.

MWM was carried out as previously described [62], with slight modifications. A circular pool was filled half-way with water at 21 °C. The maze was divided into four imaginary and equal quadrants: north (N), south (S), west (W) and east (E). A circular and transparent platform was submerged 1 cm below the surface, in the middle of one of the quadrants. The water in the pool was colored with white paint (Faber Castell Tempera Fun Paint), rendering it opaque. Visual cues were placed equidistantly in the walls of the room. In these conditions, the animals must use distal cues (extra-maze) to aid in the spatial localization of the platform. The test was composed of 4 trials per day. In each trial a semi-random start position is used, hence all positions are chosen only one time (e.g., N, W, S, E). After each trial, mice were placed in a cage with a heating pad before being returned to their home cages. During spatial acquisition, mice were placed in the pool in the start position and allowed to swim. The trial ended when mice reached the platform and stayed on it for ten seconds. If this did not occur, then the test was finished after 60 s and the mice were guided to the platform and placed on it for ten seconds. Latency to reach the platform is recorded and used to create a learning curve. The acquisition phase is finished when the average control latency to reach the platform is less than 20 s. In the probe phase, performed 24 h after the last day of acquisition, the platform was removed. Then, mice were put in the position in which they had a faster latency to reach the platform the day before and allowed to explore for 1 min. Reference memory was evaluated by the number of crossings in the site where the platform was located during the acquisition phase. Mice search strategies were evaluated using the Any Maze Software. Videos of the probe trial were analyzed, and a path was created from the start of the trial until the first time that mice crossed the site where the platform was located. Based on the path created, hippocampal-dependent or non-hippocampal-dependent strategies were assigned to each mouse, as previously described [31,62].

The open field test was carried out as previously described [62]. Mice were put in the center of a 38 × 38 cm grey acrylic box and allowed to explore the arena for 5 min. To assess locomotion, we recorded the total distance travelled (in meters) and the maximum speed they reached. Anxiety-like behavior was inferred from the time spent in the center of the open field [30,83].

### 4.4. Slice Electrophysiology

In vitro electrophysiology in acute slices from the dorsal hippocampus, prefrontal cortex (PFC) and amygdala was performed as previously described [84]. Briefly, after sacrifice by cervical dislocation, the brain was quickly removed and submerged in ice-cold, oxygenated (95% O_2_, 5% CO_2_) artificial cerebrospinal fluid (ACSF; in mM: 124.0 NaCl, 4.4 KCl, 1.0 Na_2_HPO_4_, 25.0 NaHCO_3_, 2.0 CaCl_2_, 1.0 MgCl_2_, 10.0 glucose). Coronal slices containing the prelimbic medial prefrontal cortex (PFC, 300 μm-thick) and horizontal slices containing the lateral amygdala (400 µm-thick) were cut using a Leica Vibratome (Leica, Wetzlar, Germany). Dorsal hippocampal slices (400 µm-thick) were obtained using a McIlwain tissue chopper. Slices were then placed in a holding chamber with oxygenated ACSF at 32–34 °C to recover at for at least 1 h prior to recording, when they were transferred to a submerged recording chamber and superfused with oxygenated ACSF kept at 30.5 °C, at a flow rate of 3 mL/minute.

The placement of stimulation and recording electrodes was done as described before [84]. In the hippocampus, the stimulating bipolar concentric electrode was placed in the proximal CA1 stratum radiatum for the stimulation of the Schaffer fibers and the recording electrode, filled with 4 M NaCl (2–5 MΩ resistance), was placed in the CA1 stratum radiatum targeting the distal dendrites of pyramidal neurons; in the amygdala, the bipolar concentric stimulation electrode and the recording electrode were placed in the lateral nuclei of the amygdala; in the PFC, the bipolar concentric stimulation electrode was placed in layer II/III and the recording electrode was placed in layer V. The stimulation was performed using either a Grass S44 or a Grass S48 square pulse stimulator (Grass Technologies, Carlow, Ireland) or a Digitimer DS3 stimulator (Digitimer LTD, Welwyn Garden City, UK), with rectangular pulses of 0.1 millisecond applied every 15–20 s. After amplification (ISO-80, World Precision Instruments, Hitchin, UK; or AxoPatch 200B amplifier, Axon Instruments, San Jose, CA, USA), the recordings were digitized (BNC-2110, National Instruments, Austin, TX, USA, or Digidata 1322A, Axon Instruments), averaged in groups of 3–4 and analyzed using WinLTP software [85]. The intensity of stimulation was chosen between 40–50% of maximal field excitatory postsynaptic potential (fEPSP; in the hippocampus) or population spike (PS) response (in the amygdala and prefrontal cortex), determined based on input/output curves in which stimulation of increasing intensity was given until the maximum fEPSP slope or PS amplitude was reached. Long-term potentiation (LTP) was induced by high-frequency stimulation (100 Hz for 1 s in hippocampal synapses; 300 pulses at 100 Hz, 3 min duration, five times, every 3 min in PFC synapses; 3 trains of pulses of 100 Hz for 1 s, delivered with a 5 s inter-train interval in the lateral amygdala). LTP was quantified as the percentage change between two values: the average slope or amplitude of the 10 averaged potentials taken between after LTP induction (between 50–60 min in the hippocampus and amygdala and between 35–45 min in the prefrontal cortex) in relation to the average slope of the fEPSP or the PS amplitude measured during the 10 min that preceded LTP induction.

### 4.5. Preparation of Synaptosomes and Total Protein Extracts from the Cerebral Cortex

Synaptosomes (purified synapses) and total extracts were prepared as previously described [86]. Briefly, after sacrifice, the brains were dissected on an ice-cold ACSF (same composition as above) or a sucrose solution (containing 0.32 M sucrose, 1 mM EDTA-Na, 10 mM HEPES and 1 mg/mL bovine serum albumin (BSA), pH of 7.4). The cerebral cortex was collected and stored at −20 °C until use.

Synaptosomes were prepared as previously described [87]. The cortex was homogenized in 10 mL of the sucrose solution at 4 °C. The mixture was then centrifuged at 3000× *g* for 10 min at 4 °C. The supernatant was collected and centrifuged again at 14,000× *g* for 12 min at 4 °C. The pellet was resuspended in 1 mL of a 45% (*v*/*v*) Percoll solution (45% *v*/*v* Percoll and NaCl 0.067 M, pH 7.4, volume set with Krebs HEPES Ringer buffer—KHR; containing in mM: 140 NaCl, 1 EDTA-Na, 10 HEPES, 5 KCl and 5 glucose, pH 7.4). The mixture was then centrifuged at 16,400× *g* for 2 min at 4 °C. The top layer, which contains the synaptosomes, was collected, resuspended in 2 mL of KHR solution and centrifuged at 16,400× *g* for 2 min at 4 °C. The pellet was resuspended in 250 µL of the same KHR solution and stored at −20 °C until use. To prepare for Western blot, the samples were centrifuged at 16,400× *g* for 2 min at 4 °C. The pellets were then resuspended in radioimmunoprecipitation assay buffer (RIPA; Tris-HCl 50 mM pH 7.4, NaCl 150 mM, IGEPAL (NP-40) 1%, sodium deoxycholate 0.5%, EDTA 1 mM and SDS 0.1%) with 1 mM dithiothreitol (DTT), 1 mM phenylmethylsulfonyl fluoride (PMSF), 0.001% CLAP protease inhibitor cocktail (Sigma, St. Louis, MO, USA), PhosSTOP™ phosphate inhibitors (Roche, Basel, Switzerland) and stored at −20 °C until use.

Cerebral cortical tissue was weighted, and 10 mg were used for the preparation of total protein extracts. The tissue was homogenized in RIPA buffer with 1 mM PMSF and 1 mM DTT. The homogenate was placed at 4 °C with constant agitation for 2 h, followed by a centrifugation at 13,000× *g* for 20 min at 4 °C. The supernatant was collected and stored at −20 °C until use. The determination of protein concentration was assessed with the Pierce™ BCA Protein Assay Kit (Pierce™, Thermo Scientific™, Waltham, MA, USA).

### 4.6. Western Blotting

Western blot analysis was carried out essentially as previously described [88]. Briefly, synaptosomes and total protein extracts were resuspended in sample buffer 6× (Tris 500 mM, DTT 600 mM, SDS 10%, glycerol 30% and bromophenol 0.012%) and heated for 20 min at 70 °C for denaturation. Samples (15, 30 or 60 µg of protein) were loaded on a 10% or 15% SDS-PAGE gel, with a 4% stacking gel and electrophoresed, after which, the contents of the gel were electro-transferred to a nitrocellulose membrane. The membranes were incubated for 1 h at room temperature (RT) with agitation with 5% skim milk in TBS-T or 5% BSA in TBS-T. Following blocking, the membranes were incubated (with agitation) overnight at 4 °C with the primary antibodies diluted in TBS-T with 1% skim milk or 5% BSA: mouse anti-SNAP-25 (1:10,000, Sigma S5187), mouse anti-PSD-95 (1:10,000, Cell signaling 3450), mouse anti-synaptophysin (1:10,000, Sigma S5768), mouse anti-syntaxin (1:10,000, Sigma S0664), rabbit anti-sirtuin 1 (1:1000, Cell signaling 2028, Danvers, MA, USA), rabbit anti-sirtuin 3 (1:1000, Cell Signaling, 2627), rabbit anti-sestrin 2 (1:2000, ProteinTech, 21346-1-AP, Chicago, IL, USA) and rabbit anti-GAPDH (1:10,000, Abcam AB9485, Cambridge, UK). The next day, membranes were washed with TBS-T 3 times for 10 min at RT with agitation and incubated with the secondary antibodies diluted in 1% skim milk in TBS-T or 5% BSA in TBS-T for 2 h, at RT, with agitation. The secondary antibodies used were conjugated with peroxidase for detection (1:5000, anti-mouse IgG and anti-rabbit IgG, Thermo Fischer, 31432 and 31462, respectively). Membranes were then washed with TBS-T 3 times for 10 min and then incubated with an enhanced chemiluminescence (ECL) kit (Pierce™ Thermo Scientific), a West Pico kit (SuperSignal™ Thermo Scientific, Waltham, MA, USA) or a Immobilon^®^ Forte (Millipore, Burlington, MA, USA) solution, that are substrates of peroxidase. Detection was analyzed using a ChemiDoc (BioRad, Hercules, CA, USA) and quantification was performed using the ImageLab software. After striping and reprobing, the levels of the protein of interest were calculated by applying a ratio between the density of the protein of interest and the density of glyceraldehyde 3-phosphate dehydrogenase (GAPDH). GAPDH is considered a housekeeping protein, as its density is not altered between samples of control and samples of interest, which makes it a reliable loading control and normalization protein [89].

### 4.7. Statistics

Statistical analysis was performed using GraphPad Prism Software version 8.1.1. (GraphPad Software, La Jolla, CA, USA). Results are presented as mean ± standard error of mean (SEM). Significance level was set for *p* value < 0.05 in all tests. Unpaired Student’s *t*-test was used for comparisons between two independent groups. For comparisons between two or more groups a two-way analysis of variance (ANOVA) was used followed by a post hoc Sidak’s multiple comparison test or a one-way ANOVA to evaluate the effects of different drugs on LTP. A chi-square test was used for the analysis of search strategies in the MWM test. Each result was analyzed for outlier values, by a ROUT method with a Q = 1%, and any outlier detected was excluded from the results.

## Figures and Tables

**Table 1:** Summary of the behavioral profile of 9-month-old female APP/PS1 mice.

	Summary of Genotype Comparison	APPswe/PS1dE9 vs. WT
Behavioral Test		
Morris water maze	latency to find platform	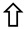
	platform crossings	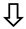
	thigmotaxis	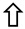
	search strategy	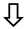 hippocampal
Fear conditioning	acquisition—day 1	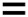
	context freezing—day 2	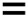
	tone freezing—day 3	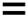
	habituation freezing—day 4	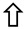
Open field	total distance	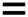
	maximum Speed	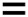
	% time in the center	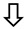

## Data Availability

Data can be made available upon reasonable request.
